# Histone Deacetylase 5 Is an Early Epigenetic Regulator of Intermittent Hypoxia Induced Sympathetic Nerve Activation and Blood Pressure

**DOI:** 10.3389/fphys.2021.688322

**Published:** 2021-05-17

**Authors:** Ning Wang, Ying-Jie Peng, Xiaoyu Su, Nanduri R. Prabhakar, Jayasri Nanduri

**Affiliations:** Institute for Integrative Physiology, Center for Systems Biology of O_2_ Sensing, The University of Chicago, Chicago, IL, United States

**Keywords:** intermittent hypoxia, obstructive sleep apnea, lysine deacetylase, HDAC5, histone 3, HIF-1, hypertension

## Abstract

Intermittent hypoxia (IH) is a hallmark manifestation of obstructive sleep apnea (OSA). Long term IH (LT-IH) triggers epigenetic reprogramming of the redox state involving DNA hypermethylation in the carotid body chemo reflex pathway resulting in persistent sympathetic activation and hypertension. Present study examined whether IH also activates epigenetic mechanism(s) other than DNA methylation. Histone modification by lysine acetylation is another major epigenetic mechanism associated with gene regulation. Equilibrium between the activities of histone acetyltransferases (HATs) and histone deacetylases (HDACs) determine the level of lysine acetylation. Here we report that exposure of rat pheochromocytoma (PC)-12 cells to IH *in vitro* exhibited reduced HDAC enzyme activity due to proteasomal degradation of HDAC3 and HDAC5 proteins. Mechanistic investigations showed that IH-evoked decrease in HDAC activity increases lysine acetylation of α subunit of hypoxia inducible factor (HIF)-1α as well as Histone (H3) protein resulting in increased HIF-1 transcriptional activity. Trichostatin A (TSA), an inhibitor of HDACs, mimicked the effects of IH. Studies on rats treated with 10 days of IH or TSA showed reduced HDAC activity, HDAC5 protein, and increased HIF-1 dependent NADPH oxidase (NOX)-4 transcription in adrenal medullae (AM) resulting in elevated plasma catecholamines and blood pressure. Likewise, heme oxygenase (HO)-2 null mice, which exhibit IH because of high incidence of spontaneous apneas (apnea index 72 ± 1.2 apnea/h), also showed decreased HDAC activity and HDAC5 protein in the AM along with elevated circulating norepinephrine levels. These findings demonstrate that lysine acetylation of histone and non-histone proteins is an early epigenetic mechanism associated with sympathetic nerve activation and hypertension in rodent models of IH.

## Introduction

Obstructive sleep apnea (OSA) is a widespread breathing disorder affecting 10–15% of adult population (Peppard et al., 2013). OSA is defined as periodic disruption of air flow during sleep either due to complete (apneas) or partial collapse (hypopnea) of the upper airway. Population based studies showed high prevalence of essential hypertension with strong correlation to severity of OSA (Morrell et al., 2000; Nieto et al., 2000).

Intermittent hypoxia (IH), a hallmark manifestation of OSA, is a major contributing factor to hypertension (reviewed in Prabhakar et al., 2020). Rats treated with long-term-IH (30 days; LT-IH) exhibit hypertension and heightened sympathetic nerve activity persisting even after a 30-day recovery in room air (Nanduri et al., 2017). The long-lasting blood pressure elevation evoked by LT-IH is reminiscent of treatment-resistant hypertension reported in a subset of OSA patients. LT-IH evoked long-lasting hypertension was due to persistent elevation of ROS caused by epigenetic suppression of anti-oxidant enzyme genes by DNA methylation (Nanduri et al., 2017).

Lysine acetylation of histones (H2A, H2B, H3, and H4) is another important epigenetic mechanism which regulates gene transcription by altering chromatin structure. Hyper-acetylation activates, while hypo-acetylation represses transcriptional activation (Grunstein, 1997; Audia and Campbell, 2016). Lysine acetylation is a dynamic process involving opposing activities of histone/lysine acetyl transferases (HAT) and histone deacetylases (HDAC’s) (Yang and Seto, 2007). A recent study reported that chronic stress decreases HDAC activity, leading to increased histone acetylation and transcriptional activation of target genes (Renthal et al., 2007). Given that IH is a form of chronic stress, whether lysine acetylation resulting from reduced HDAC activity participate in gene activation by IH has not been investigated.

In addition to histones, lysine acetylation of non-histone proteins like transcription factors also control gene transcription (Park et al., 2015). Hypoxia inducible factor (HIF-1) is a master regulator for gene transcription during hypoxia (Semenza et al., 1998). Studies on tumor cell lines showed that HDAC1 and HDAC3 positively regulate HIF-1α stability while HDAC4 and HDAC5 increase HIF-1-dependent transcriptional activation (Kim et al., 2007; Seo et al., 2009; Geng et al., 2011). Our earlier studies showed that mice partially deficient in HIF-1α, the O_2_ regulated subunit of the HIF-1 complex, exhibit remarkable absence of IH-evoked elevation of ROS, sympathetic activation and hypertension (Peng et al., 2006). Moreover, HIF-1 is a potent activator of pro-oxidant enzyme genes including NADPH oxidases (NOX) (Yuan et al., 2011; Wang et al., 2020). Given that HDACs regulate the stability of HIF-1α as well as HIF-1 activity (Qian et al., 2006; Kim et al., 2007; Seo et al., 2009), we hypothesized that IH reduces HDAC activity and stabilizes HIF-1α through lysine acetylation leading to HIF-1-dependent transcriptional activation involving lysine acetylation of histone proteins. These possibilities were first tested in rat pheochromocytoma (PC)-12 cells and then further validated in two rodent models of OSA, namely rats treated with IH and OSA-exhibiting hemeoxygenase (HO)-2 null mice (Peng et al., 2018).

## Materials and Methods

Experimental protocols were approved by the Institutional Animal Care and Use Committee (IACUC) of the University of Chicago and were performed on adult Sprague Dawley rats (SD; 2–4 months age, approved protocol #71811), age and gender matched wild-type (WT; C57BL6) and HO-2 null mice (6–8 months age, approved protocol #71810).

### Exposure of Rats to Intermittent Hypoxia (IH)

The protocols for exposing rats to IH were essentially the same as described previously (Peng et al., 2006). Briefly, conscious rats were placed in a specialized chamber and exposed to alternating cycles of hypoxia (15 s of ∼5% O_2_ at nadir followed by 5 min of room air, 8 h/day) for 10 days. Parallel experiments were performed on rats exposed to alternating cycles of room air (control) in an identical chamber. The duration of the gas flow was regulated by timer-controlled solenoid valves. Ambient O_2_ and CO_2_ levels in the chamber were continuously monitored and the CO_2_ levels were maintained at ∼0.1%.

### Exposure of Rat Pheochromocytoma (PC)-12 Cells to IH

PC12 cells (original clone from Dr. Lloyd A. Greene, Columbia University, NY, United States) (Greene and Tischler, 1976) were cultured in Dulbecco’s modified Eagle’s medium (DMEM) supplemented with 5% fetal bovine serum (FBS), 10% horse serum under 10% CO_2_ and 90% room air (20% O_2_) at 37°C (Yuan et al., 2005). Experiments were performed on cells serum-starved overnight in DMEM medium. Cells were exposed to *in vitro* IH (alternating cycles of 1.5% O_2_ for 30 s followed by 20% O_2_ for 5 min at 37°C) as described (Yuan et al., 2004). Ambient O_2_ levels in the IH chamber were monitored by an O_2_ analyzer (Alpha Omega Instruments, Huston, TX). In the experiments involving treatment with drugs, cells were pre-incubated for 30 min prior to and during IH exposure with either drug or vehicle.

### Measurement of Blood Pressure (BP) and Plasma Norepinephrine

BP was measured in conscious rats between 09.00 and 11.00 a.m. by tail-cuff method using a non-invasive BP system (IITC Life Science Inc., CA, United States). Blood samples were collected in heparinized vials (30 IU/ml) from rats/mice anesthetized with urethane (1.2 g/kg, IP). Plasma was separated by centrifugation and stored at −80°C. Plasma norepinephrine (NE) levels were determined by high pressure liquid chromatography (HPLC) combined with electrochemical detection using dihydroxybenzylamine hydrobromide (DHBA; Sigma–Aldrich) as an internal standard. The NE levels were corrected for recovery loss and expressed as nanograms per milliliter of plasma calculated from an NE standard curve (Peng et al., 2006).

### Measurement of Breathing

Breathing was monitored by whole-body plethysmography in conscious mice at ambient temperature of 25 ± 1°C as described (Peng et al., 2018). Apneas were scored by two individuals who were blinded to genotype. Number of apneas (defined as cessation of breathing for more than the duration of three breaths) per hour was analyzed and presented as the apnea index. Sighs, sniffs and movement-induced changes in breathing were excluded in the analysis.

### Measurement of HDAC, SIRT, and NOX Activity

HDAC and Sirtuin (SIRT) activities were measured in nuclear lysates using colorimetric Epigenase HDAC activity and SIRT activity kit, respectively (Epigentek, Farmington, NY, United States). Briefly, 5 μg of nuclear extract (from two rat adrenal medullae (AM) and 4 mice AM) were added to a plate coated with acetylated histone HDAC or SIRT substrate. Active HDAC or SIRT binds and deacetylates H3 which is recognized with a specific antibody and measured by reading the absorbance at 450 nm. The activity is directly proportional to the OD intensity measured and expressed as ng/min/mg protein. NADPH oxidase activity in the membrane-enriched protein fractions (50 μg: two rat AM and 4 mice AM) were measured by superoxide dismutase-inhabitable rate of cytochrome c reduction, and reading the absorbance at 550 nm. NADPH oxidase activity was calculated based on the extinction coefficient [21 mmol/(L)] per cm, and is expressed as nmol/min/mg protein (Khan et al., 2011).

### Immunoblot and Immunoprecipitation Assays

Nuclear extracts of PC12 cells were prepared using nuclear extraction kit (Active Motif, Carlsbad, CA, United States). Briefly, cells (1 × 10^6^) were homogenized in 200 μl of hypotonic buffer provided in the kit and centrifuged at 850 *g* for 10 min at 4°C. The pellet was re-suspended in 200 μl of hypotonic buffer and a small sample was checked under the microscope to verify cells were efficiently lysed and nuclei were released. The suspension was centrifuged at 14,000 *g* for 30 s. The nuclear pellet was re-suspended in 40 μl of complete lysis buffer provided and fractionated by polyacrylamide-SDS gel electrophoresis. Immunoblots were probed with either HIF-1α (1/1,000 dilution; #NB100-479, Novus Biologicals, Centennial, CO), HDAC1-5 (1/2000; #5356, #5113, #3949, #7628, and #20458, respectively) and H3 (1/4,000; #4499) (Cell signaling, Danvers, MA), TBP (1/1,000; Tata binding protein; #ab51841, Abcam) or tubulin (1/10,000; #T6199, Sigma, St. Louis, MO, United States) antibodies followed by corresponding HRP-conjugated secondary antibody detected by Clarity Western ECL substrate kit (Bio-Rad, Hercules, CA, United States). Immunoblots were scanned and quantified using an Odyssey Fc imaging system (LI-COR, Lincoln, NE, United States). For analysis of HIF-1α acetylation, PC12 cells transfected with FLAG-tagged HIF-1α plasmid were exposed to IH. Cell lysates were immunoprecipitated with anti-FLAG antibody (#1804, Sigma, St. Louis, MO, United States) and the immunoprecipitates were analyzed by acetylated lysine (1/500, #SC-32268, Santa Cruz, Dallas, TX, United States) antibody as described above. Rat/mice adrenal medulla cell extracts were prepared in RIPA buffer (phosphate buffer, pH 7.4 containing 150 mM NaCl, 1% triton X-100, 1% sodium deoxycholate, 0.1% SDS, 5 mM EDTA, and protease inhibitor cocktail) and analyzed using automated capillary electrophoresis size-based separation followed by immunoassay using the WES System from Protein Simple according to the manufacturer’s instructions. Data were analyzed with Compass software (Protein Simple). Primary antibodies against the following proteins were used at a dilution of 1/50 for immunoblot assays: HIF-1α (#NB100-123, Novus Biologicals, Centennial, CO). HDAC5 (#ab20458, Cell Signaling Danvers, MA, United States). Sample loading variability was normalized to tubulin (#T6199, Sigma, St. Louis, MO, United States) antibody. Acetylated H3 and NOX4 protein expression was analyzed in AM cell lysates by polyacrylamide-SDS gel electrophoresis and immunoblots were probed with acetylated lysine antibody (1/500, #SC-32268, Santa Cruz, Dallas, TX, United States) or NOX4 antibody (1/1,000; NB110-58851, Novus Biologicals, Centennial, CO, United States).

### qRT-PCR (Quantitative Reverse Transcription -PCR)

Total RNA was isolated with TRIZOL (Invitrogen), and cDNA was synthesized using the iScript cDNA synthesis kit (Bio-Rad). qRT- PCR was performed using SsoFast EvaGreen (Bio-RAD) as a fluorogenic binding dye. Housekeeping genes 18S RNA and β-actin were included as quantitation controls. Changes in target mRNA expression was calculated based on the Δ(ΔCt) method as described previously (Nanduri et al., 2009). Primer sequences used for real-time PCR amplification were: Rat NOX4; (NM_053524.1): Forward; CGGGTGGCTTGTTGAAGTAT; Reverse; TGGAACTTGGGTTCTTCCAG, Mouse NOX4; (NM_015760.5): Forward: TGTTGGGCCTAGGATTGTGTT; Reverse: AGGGACCTTCTGTGATCCTCG, 18S (NR_003278): Forward; CGCCGCTAGAGGTGAAATTC; Reverse; CGAACCTCCGACTTTCGTTCT. β-actin (NM_031144.3): Forward; AGG CCC AGA GCA AGA GAG G; Reverse; TAC ATG GCT GGG GTG TTG AA.

### Transient Transfections and HRE Reporter Gene Assay

PC12 cells were transiently transfected with overexpression plasmids pHDAC5 and pHDAC3 (addgene) or with control vector plasmid pcDNA using TransIT-2020 (Mirus) transfection reagent. Briefly, cells were plated in 60 mm tissue culture plates at a density of 2 × 10^6^ cells/plate in serum containing growth medium. After 24 h, DNA-TransIT reagent in the ratio of 1:3 was added. After 36 h, serum starved cells were exposed to IH. To analyze HIF-1 transcriptional activity, cells were co-transfected with two reporter plasmids: p2.1, which contains firefly luciferase coding sequences downstream of a basal SV40 promoter and a 68 bp hypoxia response element (HRE) from the human *EPO1* gene (Semenza et al., 1996) and *Renilla* Luciferase Control Reporter Vector (pRL). The Bright-Glo^TM^ Luciferase Assay System (Promega, Madison, WI, United States) was used to measure luciferase activity in nuclear cell lysates and normalized to protein content measured by Bio-Rad protein assay kit (Bio-Rad, Richmond, CA, United States).

### Data Analysis

Data is expressed as mean ± SEM from mice (8–12 animals per group) and 3–5 independent cell culture experiments. Statistical analysis was performed by analysis of variance (one-way ANOVA with *post-hoc* Turkey HSD test) and Mann-Whitney test. *p* < 0.05 were considered significant.

## Results

### IH Decreases HDAC Enzyme Activity

**FIGURE 1:**
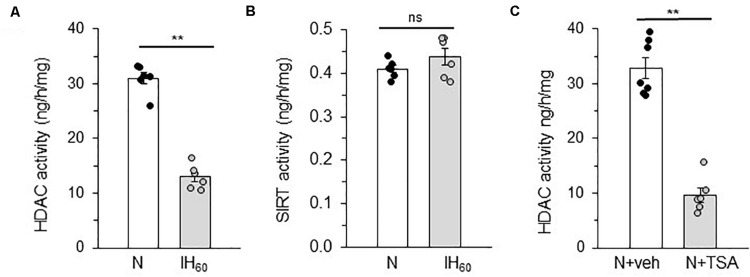
Effect of IH on HDAC and SIRT activity in PC12 cells treated with 60 cycles of IH (IH_60_) or HDAC inhibitor Trichostatin A (TSA). **(A,B)** HDAC and SIRT activity in nuclear lysates from room air (N) and IH-exposed (IH_60_) PC12 cells. **(C)** HDAC activity in nuclear lysates from PC12 cells treated with TSA (HDAC inhibitor; 50 nM) for 7 h in room air (N) (mean ± SEM; *n* = 4–6). **p ≤ 0.05; ns = not significant (*p* > 0.05) as determined by Mann-Whitney test.

Pheochromocytoma (PC) 12 cells cultures were chosen because experiments require transient transfection protocols for reporter gene assays and ectopic expression of proteins can be best performed in cell cultures rather than tissues from rats (Prabhakar et al., 2012). HDACs comprise of four classes of proteins (Haberland et al., 2009), including Class I (HDACs 1, 2, 3, and 8), Class II (HDACs 4, 5, 6, 7, 9, and 10), Class III (NAD^+^-dependent sirtuins) and Class IV (HDAC11). The enzyme activities of HDAC and Sirtuins (SIRT) were determined in cells treated with 60 cycles of IH (IH_60_) or alternating cycles of room air (N) which served as controls. Cells treated with IH_60_ showed reduced HDAC activity, whereas SIRT activity was unaltered (Figures 1A,B). Treating cells with 50 nM Trichostatin A (TSA), which is an inhibitor of HDACs but not SIRTs (Finnin et al., 1999; Bertrand, 2010), mimicked the effects of IH by markedly reducing HDAC activity compared to vehicle treated controls (Figure 1C).

### IH Reduces HDAC5 and HDAC3 Proteins

**FIGURE 2:**
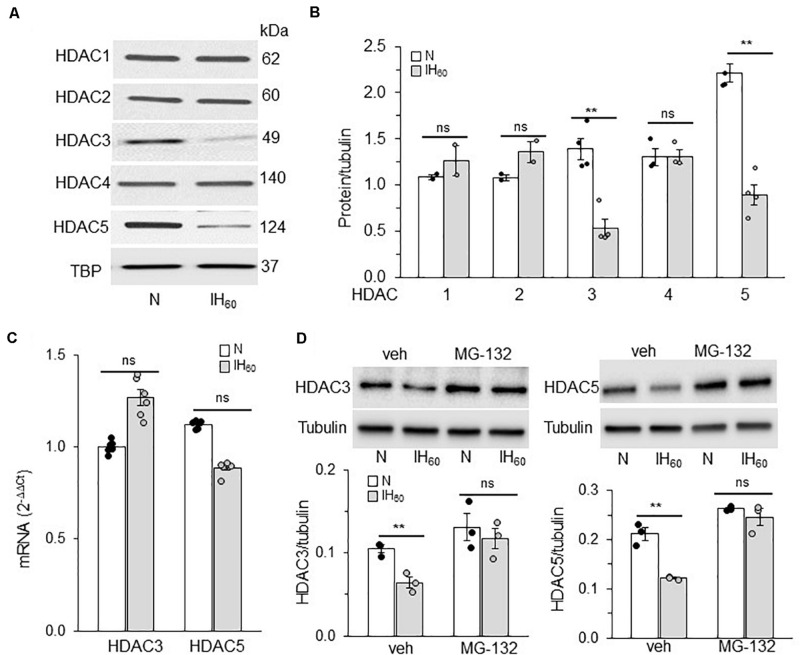
Effect of IH on HDAC proteins and mRNA expression. **(A)** Representative example of an immunoblot showing HDAC1-5 protein expression and Tata binding protein (TBP) as a loading control in nuclear lysates from room air (N) and IH-exposed PC12 cells. **(B)** Quantitative analysis of the blots by ImageJ. **(C)** HDAC3 and HDAC5 mRNA expression by qRT-PCR. **(D**) *Top panel:* HDAC3 and HDAC5 protein expression in cells treated with vehicle or MG132 (proteasomal inhibitor; 5 μM) and exposed to IH. *bottom panel:* Quantitative analysis of the blots (mean ± SEM; *n* = 3–4) by Image Studio by Odyssey Fc imaging system. **p ≤ 0.05; ns = not significant (*p* > 0.05) as determined by One-way ANOVA with *post-hoc* Turkey HSD test.

We next determined whether reduced expression of HDAC protein(s) account for the decreased HDAC activity by IH. To this end, HDAC1-5 protein abundances were determined in nuclear extracts of IH treated cells by immunoblot assay. IH treated cells showed decreased levels of HDAC3 and HDAC5 proteins, whereas the levels of HDAC1, HDAC2, and HDAC4 proteins were unaltered (Figures 2A,B).

IH had no effect on HDAC3 or HDAC5 mRNA expression (Figure 2C), suggesting that reduced HDAC3 and HDAC5 proteins by IH are not due to decreased transcription. HDACs can undergo post-translational modifications leading to ubiquitin-mediated proteasomal degradation (Adenuga et al., 2009). To determine whether the effects of IH are due to increased proteasomal degradation, cells were treated with MG-132 (5 μM), a proteasomal inhibitor. As shown in Figure 2D, cells treated with MG-132 showed absence of IH-induced reduction in HDAC3 and HDAC5 proteins compared to vehicle treated cells. These data suggest that proteasomal degradation of HDAC3 and HDAC5 contribute to decreased HDAC activity in PC12 cells exposed to IH_60_.

### Reduced HDACs Contributes to HIF-1α Stability by IH Through Lysine Acetylation

**FIGURE 3:**
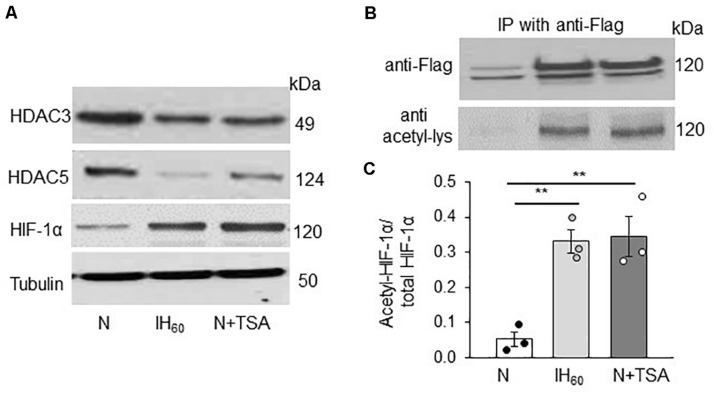
Effect of IH_60_ and TSA on HIF-1α protein and HIF-1α acetylation in PC12 cells. **(A)** HDAC3, HDAC5 and HIF-1 protein expression with tubulin as a loading control in nuclear lysates from room air (N), IH-exposed and TSA (50 nM for 7 h) treated PC12 cells; **(B)** PC12 cells transiently transfected with FLAG-tagged HIF-1 overexpression plasmids were treated with either IH_60_ or TSA and HIF-1α was immunoprecipitated with anti-FLAG antibody from nuclear extracts. Acetylation of HIF-1α was analyzed in the immunoprecipitations by immunoblots using anti-acetylated lysine antibody. HIF-1 protein was analyzed by anti-Flag antibody. **(C)** Quantitative analysis of the blots expressed as ratio of acetylated HIF-1α to total HIF-1α protein (mean ± SEM; *n* = 3). **p ≤ 0.05 as determined by One-way ANOVA with *post-hoc* Turkey HSD test.

IH stabilizes HIF-1α protein and activates HIF-1-dependent transcription in PC12 cells (Yuan et al., 2005, 2011). Given that HDACs can affect HIF-1α stability (Qian et al., 2006; Kim et al., 2007; Seo et al., 2009), we hypothesized that reduced HDAC activity by IH contributes to HIF-1α stabilization through lysine acetylation. This possibility was tested by first monitoring HIF-1α proteins in nuclear extracts of PC12 cells treated with either IH_60_ or 50 nM TSA (positive control). Decreased HDAC3 and HDAC5 protein expressions were associated with elevated HIF-1α protein in IH as well as in TSA-treated cells (Figure 3A).

We next determined whether IH or TSA-induced HIF-1α protein accumulation is associated with increased lysine acetylation of HIF-1α. Assessing endogenous HIF-1α lysine acetylation in PC12 cells was technically challenging. To circumvent this limitation, PC12 cells were transfected with a FLAG-tagged HIF-1α expressing plasmid and then treated with either IH_60_ or TSA (50 nM for 7 h). HIF-1α was immunoprecipitated with FLAG-antibody in nuclear extracts and lysine acetylation was determined using anti-acetylated lysine antibody. Cells treated with IH_60_ as well as TSA showed increased abundance of lysine acetylated HIF-1α protein compared to room air (N) or vehicle treated controls (Figures 3B,C).

### HDACs Regulate IH-Induced HIF-1 Transcription Through Histone Lysine Acetylation

**FIGURE 4:**
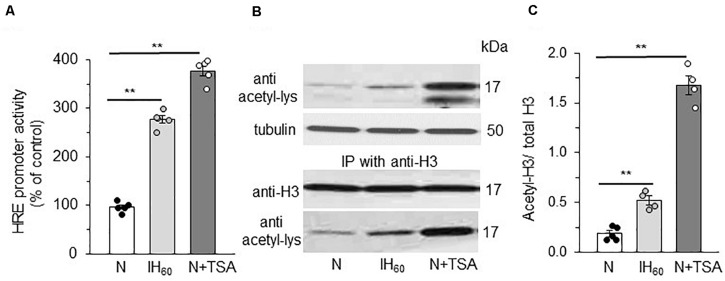
Effect of IH_60_ and TSA on HIF-1 transcriptional activity and Histone (H3) acetylation in PC12 cells. **(A)** HRE transcriptional activity in nuclear lysates of cells co-transfected with a plasmid p2.1 containing HRE upstream of SV40 promoter and luciferase coding sequence and Renilla luciferase control vector and exposed to room air (N), IH and TSA treated. **(B)** Acetylation of Histone 3 (H3) as analyzed by immunoblots with anti-acetyl lysine antibody. Acetylation of H3 was confirmed by immunoprecipitating with anti H3 antibody and the immunoprecipitations probed with anti-H3 antibody and anti-acetyl lysine antibody. **(C)** Quantitative analysis of the blots expressed as ratio of acetylated H3 to total H3 protein (mean ± SEM; *n* = 3–4). **p ≤ 0.05 as determined by One-way ANOVA with *post-hoc* Turkey HSD test.

We then asked whether HIF-1α stabilization by lysine acetylation accompanies HIF-1 transcriptional activation by IH. To this end, cells were transfected with a plasmid containing a hypoxia response element (HRE) upstream of luciferase coding sequences. Increased luciferase activity was monitored as an index of HIF transcriptional activity. Cells treated with IH_60_ as well as TSA showed robust HIF-1 dependent transcriptional activation as evidenced by marked increase in luciferase activity (Figure 4A).

Lysine acetylation of histones regulate transcription by facilitating binding of transcription factors to promoter regions of target genes through altering chromatin structure (Yang and Seto, 2003; Seto and Yoshida, 2014). We assessed whether reduced HDAC activity contributes to HIF-1 transcriptional activation by IH through increased histone lysine acetylation. This possibility was tested first by determining which histones were acetylated in PC12 cells with IH or TSA. Immunoblot assay with a pan acetylated lysine antibody detected a band around 17 kDa in IH and TSA treated cell lysates. This molecular mass corresponds to histone 3 (H3) rather than histone 4 (∼11 kD) (Figure 4B). To confirm that IH increases H3 lysine acetylation, H3 was immunoprecipitated from nuclear extracts with anti-H3 antibody and the immunoprecipitates were probed for lysine acetylation with acetylated lysine antibody. Abundance of lysine acetylated H3 protein increased in IH as well as in TSA treated cells with no change in the total H3 protein expression (Figures 4B,C). These observations establish that decreased HDAC activity increase lysine acetylation of H3 protein, which accompanies HIF-1 transcriptional activation by IH.

### HDAC5 but Not HDAC3 Regulates HIF-1 Activation by IH

**FIGURE 5:**
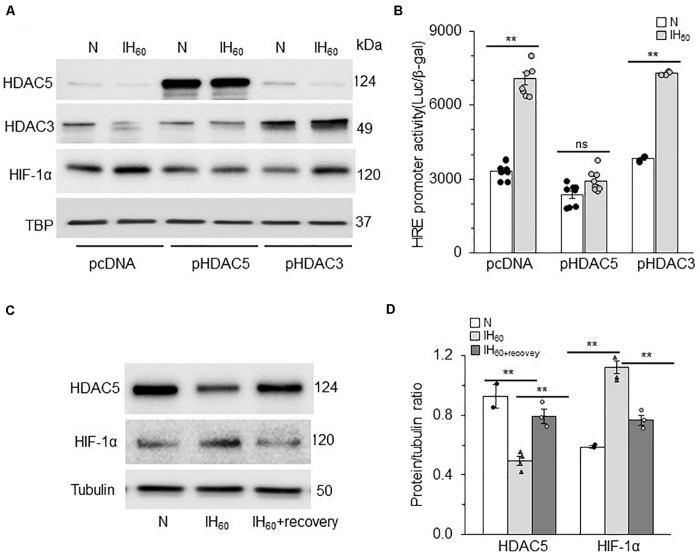
HDAC5 but not HDAC3 contributes to IH-augmented HIF-1 transcriptional activity. **(A)** Representative example of an immunoblot showing HDAC5, HDAC3, and HIF-1α protein expression with Tata binding protein (TBP) as a loading control in nuclear lysates from room air (N) and IH-exposed PC12 cells transfected with overexpression plasmids encoding either HDAC3, HDAC5, or control vector pcDNA (mean ± SEM; *n* = 3). **(B)** HRE transcriptional activity in nuclear lysates from control (N) and IH-exposed PC12 cells overexpressing HDAC3, or HDAC5 protein (mean ± SEM; *n* = 6). **p ≤ 0.05; ns = not significant (*p* > 0.05) as determined by Mann-Whitney test. **(C)** Immunoblot showing HDAC5, HIF-1α protein and acetyl-H3 protein expression with tubulin as a loading control in cell lysates from room air (N) IH-exposed (IH_60_) and IH-exposed cells after room air recovery for 16 h (IH_60__+__*recovery*_). **(D)** Quantitative analysis of the blots. **p ≤ 0.05 as determined by One-way ANOVA with *post-hoc* Turkey HSD test.

HDACs function as multimeric protein complexes with homo- and/or heterodimer structure (Yang and Seto, 2003). Given that IH decreases both HDAC3 and HDAC5 proteins, we examined the relative contribution of HDAC3 and HDAC5 to IH-evoked HIF-1 transcriptional activation. To this end, cells were transfected with over expression plasmids encoding either HDAC3 or HDAC5, and then exposed to IH_60_ or normoxia (control). Over expression of HDAC5 blocked the reduced HDAC5 expression as well as HIF-1α protein accumulation characteristic to IH (Figure 5A) with no significant effect on HDAC3 protein. On the other hand, overexpression of HDAC3 blocked IH-induced decrease in HDAC3 expression but had no effect on either IH-induced HIF-1α or HDAC 5 protein expression (Figure 5A). Furthermore, IH-evoked HRE transcriptional activity was absent in cells with ectopic expression of HDAC5 but not HDAC3 (Figure 5B). The effects of IH on HDAC5 and HIF-1α protein were reversed within 16 h after recovery of cells in room air (Figures 5C,D). These findings demonstrate that reduced HDAC5 but not HDAC3 contribute to HIF-1α stability and HIF-1 transcriptional activation by IH and that the effects of IH on the HDAC5 protein are reversible.

### HDAC Regulation of HIF-1 Activity in Rodent Models of OSA

To validate the observations in cell culture studies, the following experiments were performed on rodents. Two rodent models of IH were studied: (1) rats treated with 10 days of IH patterned after blood O_2_ profiles during OSA; and (2) mice deficient in hemeoxygenase (HO)-2, which experience IH because of high incidence of spontaneous apneas (Peng et al., 2017).

#### Rats Treated With IH

**FIGURE 6:**
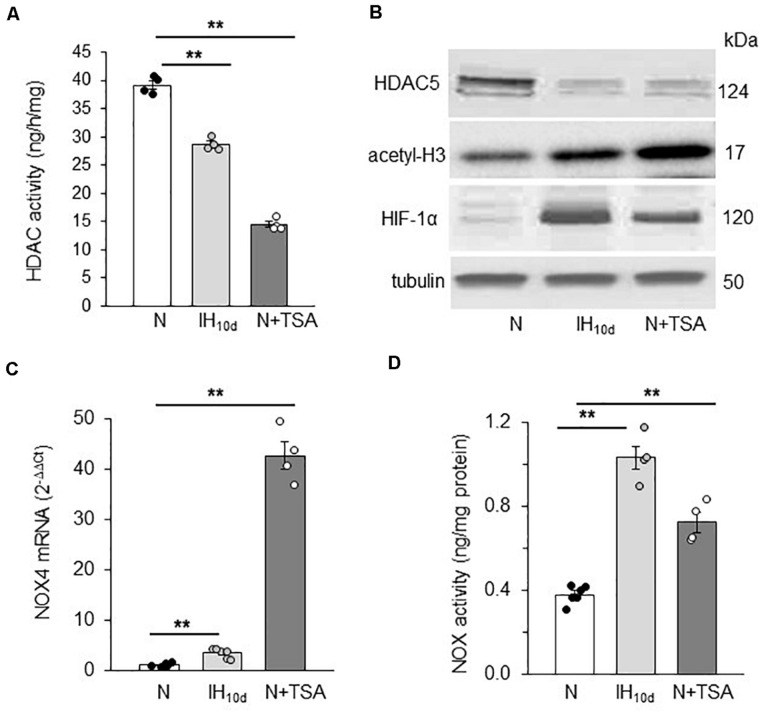
Effect of IH or TSA treatment on HDAC activity, HDAC5, HIF-1α and H3 protein, NOX 4 mRNA and NOX activity in AM of SD rats. Adult rats were either exposed to IH (IH_1__0d_) or treated with TSA (10 mg/Kg; IP) for 10 days. Control experiments were performed on rats exposed to room air (N). Cell lysates prepared from AM were analyzed for **(A)** HDAC activity, **(B)** HDAC5, acetyl-H3, HIF-1α protein expression with tubulin as loading control by immunoblots, **(C)** NOX4 mRNA expression by qRT-PCR and **(D)** NOX activity, which was analyzed in membrane protein fractions (2 a.m. from each rat pooled) as described in methods. Data are mean ± SEM from 8 rats per group. **p ≤ 0.05 as determined by One-way ANOVA with *post-hoc* Turkey HSD test.

Adult rats were treated with IH or TSA (10 mg/kg; I.P), an inhibitor of HDAC, for 10 days, the latter as a positive control for HDACs. Rats treated with alternating cycles of room air served as controls. HDAC activity, HDAC5, and HIF-1α protein as well as H3 acetylation were determined in adrenal medulla (AM) harvested from anesthetized rats. The AM tissue was chosen for two reasons: (1) PC12 cells, which were used in the above cell culture studies are of adrenal medullary chromaffin cell origin (Greene and Tischler, 1976); and (2) AM plays a critical role in IH-induced hypertension (Bao et al., 1997; Prabhakar et al., 2012). Technical limitations precluded measurements of lysine acetylation of HIF-1α protein in these experiments. HDAC activity and HDAC5 protein expressions were reduced in AM of rats treated with IH as well as TSA (Figures 6A,B). These effects were associated with increased H3 lysine acetylation, increased HIF-1α protein and elevated NOX4 mRNA, a HIF-1 target gene (Figures 6B,C). The elevated NOX4 mRNA was accompanied with increased NOX enzyme activity as compared to room air treated controls (Figure 6D).

#### Studies on HO-2 Null Mice

**FIGURE 7:**
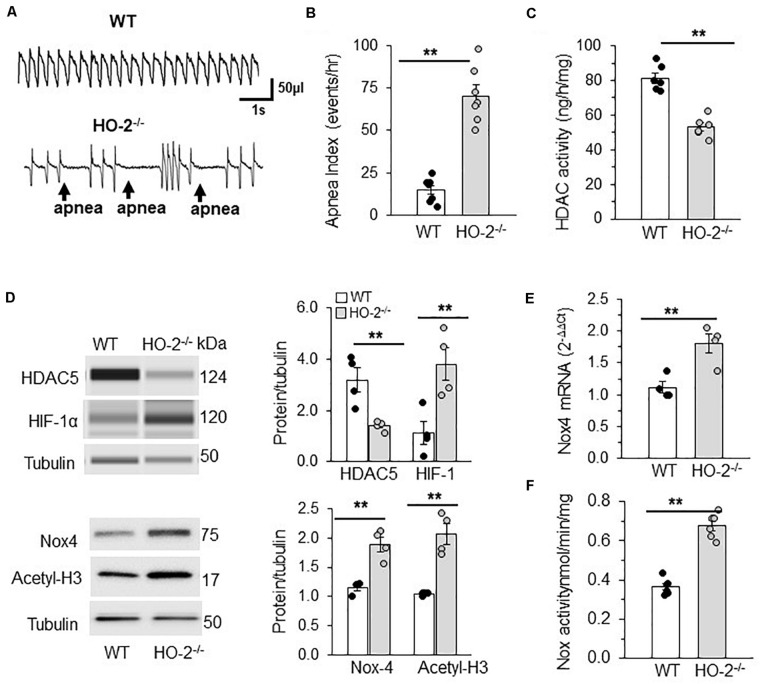
HDAC regulation of HIF-1 in rodent model of OSA. **(A)** Representative tracing of breathing in age matched (6 months) C57BL6 mice (WT) and HO-2 null mice (*HO-2^− /−^*) mice as measured by plethysmography. **(B)** Number of apneas (cessation of breathing for more than three breaths) measured per hour represented as apnea index. **(C)** HDAC activity in nuclear extracts (pooled 4 a.m. for single measurement). **(D)** Top panel: Representative immunoblot of HDAC5, HIF-1α and tubulin (loading control) protein expression as determined by the WES system from Protein simple; Nox4, Acetyl H3 and tubulin (loading control) proteins determined by SDS-PAGE gel electrophoresis. Bottom panel: Quantitative analysis of HDAC5 and HIF-1 proteins using Compass software (Protein Simple). NOX4 and acetyl H3 data was analyzed by ImageJ. **p ≤ 0.05 determined by One-way ANOVA with *post-hoc* Turkey HSD test. **(E)** Nox4 mRNA expression by qRT-PCR and **(F)** NOX activity in the membrane lysates of AM (pooled 4 a.m. for single measurement) of C57BL6 mice (WT) and *HO-2^− /−^* mice. Data are mean ± SEM from 12 mice per group. **p ≤ 0.05 as determined by Mann-Whitney test.

Mice deficient in the enzyme hemeoxygenase-2 (HO-2), exhibit sleep apnea (Peng et al., 2017). Apnea index (AI = number of apneas/h) was analyzed by recording breathing by plethysmography for 5 h in un-sedated WT and HO-2 null mice. Examples of breathing in WT and HO-2 null mice are shown in Figure 7A. WT mouse displayed stable breathing with an apnea index (AI) of 10 ± 7 apneas/hr. HO-2 null mice showed irregular breathing with a high incidence of apneas, with an AI of 75 ± 16 apneas/h (Figures 7A,B). AM from HO-2 null mice showed reduced HDAC activity and HDAC5 protein compared to WT AM (Figures 7C,D). In addition, HO-2 null AM showed higher abundance of HIF-1α protein and increased H3 acetylation with concomitant increase in NOX4 mRNA (target gene of HIF-1α), NOX4 protein and NOX activity compared to WT AM (Figures 7D–F).

### Reduced HDAC Expression Elevates Blood Pressure and Plasma NE Levels

**FIGURE 8:**
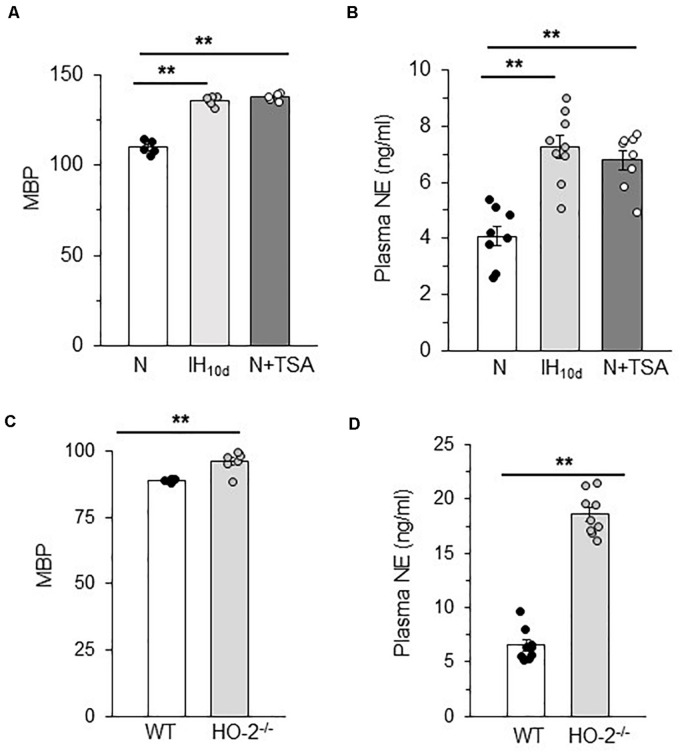
Plasma norepinephrine and BP in TSA treated rats and HO-2 null mice **(A)** Mean blood pressures (MBP) measured after 15 h of terminating IH or TSA treatment. **(B)** Plasma norepinephrine (NE) levels in rats treated with IH with or TSA. Data are mean ± SEM from 8 rats per group. **p ≤ 0.05 as determined by One-way ANOVA test. **(C)** Mean blood pressures and **(D)** plasma NE levels in age matched (6 months) C57BL6 (WT) and *HO-2^− /−^*mice. Data are mean ± SEM from eight mice per group. **p ≤ 0.05 as determined by Mann-Whitney test.

IH treated rodents exhibit elevated sympathetic nerve activity and hypertension and these responses are associated with HIF-1-dependent transcriptional activation of NOX (Prabhakar et al., 2015). Given that reduced HDAC activity by IH leads to HIF-1-dependent NOX activation, we hypothesized that these responses will be accompanied with activation of the sympathetic nervous system and elevated BP. To assess whether the systemic effects are linked to reduced HDAC activity, BP and plasma NE levels (a marker of sympathetic nerve activation) were monitored in rats treated with IH or TSA (10 mg/kg/day; 10 days; I.P). TSA treated rats exhibited elevated BP and plasma NE levels similar to IH treated rats (Figures 8A,B). Likewise, HO-2 null mice, which showed higher AI and decreased HDAC activity also showed elevated BP and plasma NE levels compared to WT mice (Figures 8C,D).

## Discussion

The present study examined the role of HDACs in HIF-1 transcriptional activation by IH. Cell culture studies showed that: (1) IH decreases HDAC activity, which was due to reduced HDAC5 and HDAC3 protein expression; (2) reduced HDAC activity stabilizes HIF-1α through lysine acetylation of the HIF-1 protein and (3) activates HIF-1-mediated transcriptional activity via histone (H)-3 lysine acetylation. Similar to PC12 cells, AM tissue from rats treated with IH and from HO-2 null mice, which exhibited a high incidence of spontaneous apneas, also showed reduced HDAC5 protein and enzyme activity while simultaneously exhibiting elevated plasma NE levels and BP. Treating control rats with TSA, a pan HDAC inhibitor, in room air mimicked the effects of IH. These findings demonstrate a hitherto uncharacterized role for HDACs in HIF-1 activation by IH leading to autonomic morbidities in rodent models of OSA.

Although both HDACs and SIRTs function as histone deacetylases (Lim et al., 2010; Geng et al., 2011), IH decreased HDAC but not SIRT enzyme activity. Furthermore, TSA, a selective inhibitor of HDACs but not SIRTs, mimicked the effects of IH in both cell culture and in rodent models of OSA. The role of SIRT in IH evoked HIF-1 activity, if any, remains to be investigated.

**FIGURE 9:**
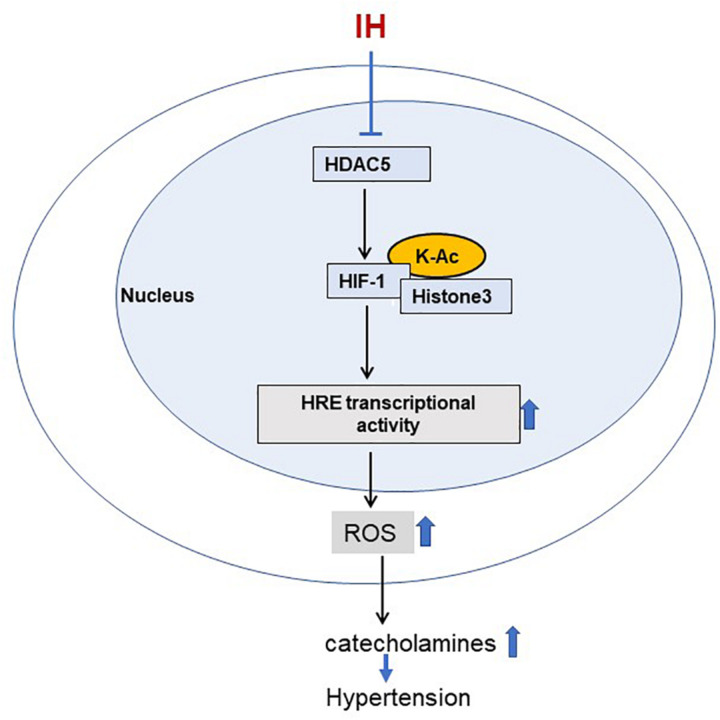
Schematic representation for the role of HDAC5 in IH induced sympathetic activation. IH-decreased HDAC activity leads to increased acetylation of HIF-1 and H3 resulting in augmented HIF-transcriptional activity of pro-oxidant enzyme NADPH oxidases. This effect is associated with elevated ROS levels leading to increased plasma catecholamines, a marker of heightened sympathetic activation, and hypertension.

Reduced HDAC activity leads to hyperacetylation, which is known to activate gene transcription (Grunstein, 1997). Consistent with this possibility, decreased HDAC activity by IH was associated with increased HIF-1-dependent gene activation, a major molecular mechanism implicated in autonomic morbidity caused by IH (Prabhakar et al., 2020). Activation by HIF-1-mediated transcription of target genes requires two steps: (1) Stabilization of HIF-1α, the O_2_ regulated subunit; and (2) transactivation of the target gene. Inhibition of prolyl hydroxylases (PHDs) is generally thought to stabilize HIF-1α in response to continuous hypoxia (Semenza, 2001). However, PHDs appear to play a less prominent role in IH-induced HIF-1α stabilization (Yuan et al., 2008). Emerging evidence suggests that additional mechanisms including acetylation-deacetylation of HIF-1α protein also contribute to HIF-1α stability (Jeong et al., 2002; Lim et al., 2010). Co-immunoprecipitation studies with nuclear extracts from IH or TSA treated PC12 cells demonstrated increased lysine acetylation of HIF-1α protein, consistent with the notion that HDACs can regulate acetylation of non-histone proteins (Park et al., 2015). Multiple acetylation sites have been identified within the HIF-1α protein, which can contribute to stabilization of the HIF-1α protein (Jeong et al., 2002; Lim et al., 2010; Geng et al., 2011; Seo et al., 2015). Lysine acetylation was shown to inhibit ubiquitination dependent proteasome–mediated degradation (Caron et al., 2005). It is therefore likely that under normoxia, HDACs keep HIF-1α in a deacetylated state, making it susceptible to proteasome degradation. During IH, decreased HDAC activity leads to increased lysine acetylation of HIF-1α, thereby enhancing the protein stability (Figure 9).

IH activated HIF-1 transcription in cell cultures as well as tissues from rodents. The increased HIF-1 transcription was accompanied with increased lysine acetylation of H3 (Figure 4B). Given that lysine acetylation of histone opens up the chromatin thereby facilitating binding of transcription factors (Seto and Yoshida, 2014), it is conceivable that H3 acetylation contributes to transcriptional activation of HIF-1 by facilitating the accessibility of HIF-1 to the promoter region of target genes such as NOX4, a possibility that requires additional studies and is beyond the scope of the current investigation.

The reduced HDAC activity by IH was accompanied with decreased HDAC5 (class II) and HDAC3 (class I) protein abundance. Because HDACs function as multimeric protein complexes (Seto and Yoshida, 2014), we anticipated that coordinated reduction of HDAC5 and HDAC3 is necessary for HIF-1 activation by IH. Contrary to our expectation, overexpression of HDAC5 alone was sufficient to block IH-induced HIF-1α protein accumulation as well as transcriptional activation. On the other hand, overexpression of HDAC3 was ineffective in altering HIF-1 activation by IH. These findings suggest that individual HDACs have distinct functions in the context of IH. However, further studies are necessary to delineate the role, if any, of HDAC3 in IH-induced physiological changes.

The reduced HDAC3 and HDAC5 protein expressions by IH were not due to reduced transcription but to increased proteasomal degradation (Figures 2A,B). HDACs can undergo post-translational modification by phosphorylation, leading to ubiquitination and proteasomal degradation (Adenuga et al., 2009). HDAC5 is known to shuttle between the nucleus and cytoplasm in a calcium- and phosphorylation-dependent manner in response to stimuli (Chawla et al., 2003; Renthal et al., 2007). It is likely that post-translational modifications involving phosphorylation play a role in reduced HDAC5 protein by IH. We have previously shown that IH increases reactive oxygen species (ROS) levels in several tissues (Peng and Prabhakar, 2003) which are known to regulate protein kinases and phosphatases (Yuan et al., 2008; Corcoran and Cotter, 2013). Whether the decreased HDAC3 and HDAC5 proteins by IH involve ROS-dependent post-translational phosphorylation modifications remains to be studied.

The current study provides important insights linking HDAC alterations, sympathetic nerve activation, and elevated BP in rodent models of OSA. How might augmented HIF-1 transcriptional activity by HDACs contribute to heightened sympathetic nerve activation and BP elevation by IH? HIF-1–dependent transcriptional activation of pro-oxidant enzyme genes such as NOX and the ensuing ROS signaling have been identified as an important molecular mechanisms underlying IH-induced sympathetic activation and BP elevation (Prabhakar et al., 2020). HIF-1-dependent NOX activation augments CA release from AM and elevates BP in IH treated rats as reported previously (Kumar et al., 2006; Kuri et al., 2007; Peng et al., 2014). Whether HDAC-dependent HIF-1 activation in addition to NOX also affects CA secretion from the AM through one or more components of the exocytosis mechanisms of adrenal medullary chromaffin cells remains to be investigated. Despite these limitations, our findings establish a pathogenic mechanism linking HDACs, HIF-1, ROS generation and cardiovascular pathology associated with IH/OSA (Figure 9).

The present study demonstrates that IH activates lysine acetylation of H3 and HIF-1α, an epigenetic mechanism that alters chromatin, thereby affecting the accessibility of DNA for transcription factors without changes in the coding sequence of DNA *per se.* We previously reported that IH triggers epigenetic re-programming of the redox state involving DNA hypermethylation (Nanduri et al., 2017). However, there are important differences between the DNA methylation and lysine acetylation by HDAC, the two epigenetic mechanisms activated by IH. These include: (1) DNA methylation is activated by long-term IH, whereas lysine acetylation resulting from reduced HDAC is initiated even with short-term IH treatment; (2) DNA methylation is linked primarily to anti-oxidant enzyme genes, whereas lysine acetylation is linked to NOX activation, which is a pro-oxidant gene; and (3) DNA methylation is linked to long lasting elevation of BP and plasma NE responses which were not reversed even after 30 days recovery in room air, whereas the systemic responses associated with lysine acetylation and reduced HDAC5 are reversed by a few hours of recovery in room air as shown in the present study (Figures 5C,D) and as reported previously (Nanduri et al., 2017). Epigenetic mechanisms involving histone post-translational modifications and DNA methylation are interrelated mechanisms. It is therefore possible that early epigenetic changes involving lysine acetylation of histone and non-histone proteins due to decreased HDAC activity may trigger DNA methylation seen with LT-IH which remains to be established.

HDACs function as transcriptional repressors or activators in health and disease (Haberland et al., 2009). For example, certain types of cancers are associated with increased expression of HDACs. Consequently, HDAC inhibitors are currently considered novel potential therapeutic interventions for alleviating cancers. On the other hand, HDACs are known to have a protective role in patients with inflammatory lung disease (Adcock et al., 2005) as well as cardiac damage (Chang et al., 2004). The current results indicate an additional unexplored role for HDACs in contributing to autonomic morbidities associated with OSA, a wide-spread breathing disorder.

## Data Availability Statement

The raw data supporting the conclusions of this article will be made available by the authors, without undue reservation.

## Ethics Statement

The animal study was reviewed and approved by the Institutional Animal Care and Use Committee (IACUC) of the University of Chicago.

## Author Contributions

JN conceived, designed the experiments, and wrote the manuscript. NW, XS, and Y-JP performed the experiments and analyzed the data. NP edited the manuscript. All authors contributed to the article and approved the submitted version.

## Conflict of Interest

The authors declare that the research was conducted in the absence of any commercial or financial relationships that could be construed as a potential conflict of interest.
